# Role of TGF-β signaling in uterine carcinosarcoma

**DOI:** 10.18632/oncotarget.3711

**Published:** 2015-03-30

**Authors:** Shailendra Kumar Dhar Dwivedi, Scott D. McMeekin, Katrina Slaughter, Resham Bhattacharya

**Affiliations:** ^1^ Department of Obstetrics and Gynecology, Stephenson Cancer Center, University of Oklahoma Health Science Center, Oklahoma, USA

**Keywords:** uterine carcinosarcoma, EMT, c-Myc, TGF beta

## Abstract

Uterine carcinosarcomas (UCS) are rare (3-4%) but highly aggressive, accounting for a disproportionately high (16.4%) mortality among uterine malignancies. Transforming growth factor beta (TGFβ) is a multifunctional cytokine that regulates important cellular processes including epithelial-mesenchymal transition (EMT). Existence of biphasic elements and a report demonstrating amplification of TGFβ at 19q13.1 prompted us to investigate the role of TGFβ signaling in UCS.

Here we demonstrated the components of TGFβ pathway are expressed and functional in UCS. TGFβ-I induced significant Smad2/3 phosphorylation, migration and EMT responses in UCS cell lines which could be attenuated by the TGFβ receptor I (TGFβR-I) or TGFβ receptor I/II (TGFβR-I/II) inhibitor developed by Eli Lilly and company. Importantly, TGFβ-I induced proliferation was c-Myc dependent, likely through activation of cell cycle. c-Myc was induced by nuclear translocation of nuclear factor of activated T cells (NFAT-1) in response to TGFβ-I. Inhibition of NFAT-1 or TGFβR-I blocked c-Myc induction, cell cycle progression and proliferation in UCS. In corroboration, mRNA levels of c-Myc were elevated in recurrent versus the non-recurrent UCS patient samples. Interestingly, in the absence of exogenous TGFβ the TGFβR-I/II inhibitor enhanced proliferation likely through non-Smad pathways. Thus, inhibition of TGFβR-I could be efficacious in treatment of UCS.

## INTRODUCTION

UCS are biphasic tumors that are highly aggressive and rare accounting for ~3-4% of all uterine cancers. By definition these tumors are composed of a malignant epithelial component, typically with high grade endometrioid or serous features [1, 2]. The sarcomatous component contains neoplastic cells with morphology homologous or heterologous to tissue within the uterus. Arising mainly in the uterus, UCS has a high rate of extra-uterine spread at diagnosis and high rate of recurrence responsible for 16.4% mortality caused by uterine malignancies [3]. Even patients with early stage disease have recurrence rates of 30-50%. Despite the improvements noted with chemotherapy, less than 30% of patients with optimally resected, stage III-IV disease, remain progression free at 3 years. For patients with bulky advanced/recurrent disease, ~15% of patients remain progression free at 2 years, and only 20% remain alive at 2 years [4, 5]. Clearly, there is a compelling need for improvement of existing treatments.

TGFβ is a multifunctional cytokine that not only regulates EMT [6], but in epithelial cells it suppresses growth and proliferation [7-9]. Contrastingly, aberrations in the TGFβ signaling regularly take place during tumorigenesis inducing the cancer cells to proliferate, invade, and metastasize beyond their tissue of origin [10-17]. Active TGFβ binds to the extracellular domain of a type II receptor, (TGFβR-II), which then recruits and activates the type I receptor subunit (TGFβR-I). This active receptor complex phosphorylates and activates the receptor-activated Smads (R-Smad), Smad2 and Smad3. Activated R-Smads then heterodimerize with the co-Smad- Smad4, and this complex translocates to the nucleus modulating specific target gene expression [18, 19]. Information regarding TGFβ signaling in UCS is extremely limited. Chiyoda et al. recently reported that acquired markers of EMT were up-regulated and the TGFβ locus was amplified in 4 out of 7 UCS patient samples [20]. Hence the presence of biphasic, epithelial and mesenchymal elements in UCS and the known role of TGFβ in regulating EMT prompted us to investigate the functional role of TGFβ and whether the TGFβR inhibitors would be efficacious in UCS.

Using patient samples and cell lines we have shown that the components of the TGFβ pathway are expressed and functional in UCS. Importantly, mRNA levels of TGFβ-I, TGFβ-II, TGFβR-I and TGFβR-II were higher in recurrent compared to the non-recurrent UCS patient samples. Using UCS cell lines we demonstrated that TGFβ-I induces significant Smad2/3 activation, cell proliferation, migration and EMT. We next evaluated the efficacy of inhibiting TGFβR-I (using LY2157299, Galunisertib currently in clinical trials for solid tumors) or TGFβR-I/II (using LY2109761) in mediating TGFβ-I induced proliferation, migration and EMT. LY2157299 and LY2109761 both inhibited Smad2/3 activation and TGFβ-I dependent migration. TGFβ-I induces NFAT-1 dependent c-Myc induction and proliferation in one UCS cell line. Interestingly, mRNA levels of c-Myc were elevated in the recurrent compared to the non-recurrent UCS patient samples. Importantly TGFβR-I inhibitor blocked TGFβ−Ι induced c-Myc expression and subsequent proliferation. Both TGFβR-I and TGFβR-I/II inhibitor blocked TGFβ−Ι induced proliferation. Remarkably in absence of exogenous TGFβ−Ι the TGFβR-I/II inhibitor dose-dependently enhanced proliferation likely through non-Smad pathways. Therefore, inhibition of TGFβR-I in UCS could be efficacious in inhibiting TGFβ-I mediated EMT, proliferation and migration, while NFAT-1 and c-Myc could be potential prognostic markers predicting poor outcome.

## RESULTS

### Components of TGFβ pathway are expressed in UCS patient tissues and cell lines

**Figure 1:**
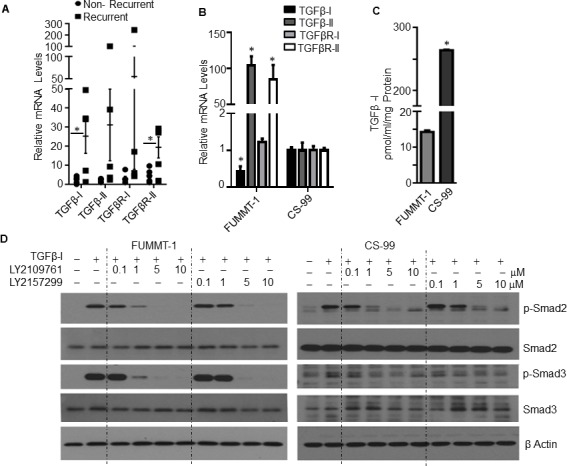
TGFβ signaling in UCS **A**. RNA was isolated from UCS patient samples and relative mRNA levels were quantified using RT-qPCR compared to non-recurrent patient sample having highest ΔCT value, Error bars represent standard error of mean. **B**. RNA was isolated from UCS cell lines and relative mRNA levels were quantified using RT-qPCR compared to CS-99. **C**. Cell free conditioned medium was collected from starved cells, TGFβ-I concentration was quantified & normalized with the total protein amount from the lysed cells. **D**. Cells were serum starved for 4h, pretreated with TGF-β receptor inhibitor subsequently treated with TGFβ-I (5 ng/ml) for 15 minutes, lysed and immunoblotted. *, *P* < 0.05 was considered significant. Error bars represent Standard Deviation (SD) except where indicated.

The biphasic nature and a report demonstrating amplification of the TGFβ locus at 19q13.1 in UCS [20] prompted us to determine whether the TGF pathway is active in UCS patient samples. To this end we performed quantitative real time PCR (RT-qPCR) with RNA isolated from 10 UCS patient tumor samples. Of the 10, 5 recurred with progression free survival (PFS) ranging between 3-7 months and 5 patients remained free of recurrence with follow-up time ranging between 5-60 months. Interestingly, the relative mRNA expression of TGFβ-I, TGFβ-II, TGFβR-I and TGFβR-II (Fig. 1A) showed a trend towards higher expression in patients whose tumor had recurred versus those that did not recur with TGFβ-I and TGFβR-II being statistically significant. These mRNA levels were also evaluated in two UCS cell lines CS-99 [21] and FUMMT-1 [22] that had been previously described to be primarily sarcomatous yet expressing certain epithelial components. With the exception of TGFβ-I; TGFβ-II, TGFβR-I and TGFβR-II were expressed at significantly higher levels in FUMMT-1 compared to CS-99 (Fig. 1B). In accordance with the mRNA expression, CS-99 secretes significantly more TGFβ-I than FUMMT-1 (263.61 ±1.36 vs 19.58±0.37 pg/ml/mg protein) (Fig. 1C).

Having established that the primary components of the TGFβ pathway were expressed in UCS patient tissues and cell lines, we next evaluated Smad signaling in response to TGFβ−Ι in these cell lines. Stimulation with TGFβ-Ι induced significant phosphorylation of Smad2 and Smad3 in both FUMMT-1 and CS-99 cell lines indicating preservation of canonical signaling in these cell lines. Since TGFβ mediated signaling was intact we next tested the efficacy of LY2157299, TGFβR-I inhibitor or LY2109761, TGFβR-I and II dual inhibitor in inhibiting TGFβ mediated Smad signaling. Both the TGFβR-I and TGFβR-I/II inhibitors decreased Smad2 and Smad3 phosphorylation in a dose-dependent manner, however at lower concentrations of 0.1-1 μM, the dual inhibitor demonstrated slightly better efficacy (Fig. 1D). These inhibitors have been developed by Eli-Lilly and company. LY2157299 (Galunisertib) is currently the only TGF-β receptor kinase inhibitor being tested in Phase II trials for glioma, pancreatic cancer and hepatocellular cancer [23].

### Effect of TGFβ on cell proliferation, migration and EMT

**Figure 2:**
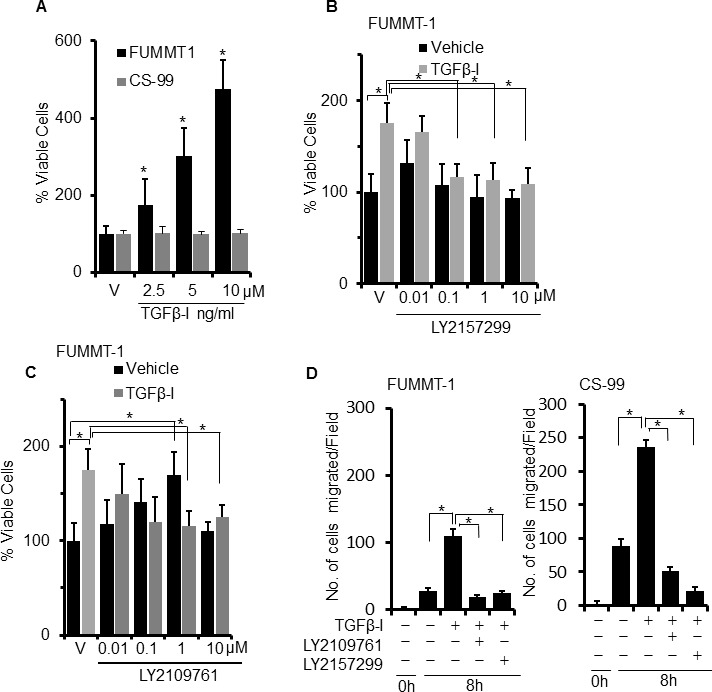
Effect of TGFβ on cell proliferation and migration **A**. Cells were serum starved and treated with TGFβ-I for 24 h, % cell viability was determined using the MTS assay. **B**. and **C**. FUMMT-1 cells were serum starved for 4h, pretreated with LY2157299 (B) or LY2109761 (C) subsequently treated with TGFβ-I (5 ng/ml) for 24 h, % cell viability was determined using MTS assay. **D**. Scratch wounds were made to starved, confluent monolayers of FUMMT-1 and CS-99 cells, pretreated with TGFβ receptor inhibitors (5 μM for 1 h) followed by 8h TGFβ-I (5 ng/ml) treatment. Micrographs were captured just after treatment and after 8 h treatment. Number of migrated cells were counted and plotted. *, *P* < 0.05 was considered significant. Error bars represent SD.

Since TGFβ is a multifunctional cytokine that not only regulates EMT, but can also suppress or induce proliferation and migration in cell type specific manner, we next evaluated the effect of TGFβ−Ι on cell proliferation using the MTS assay. TGFβ-I induced significant dose-dependent proliferation in FUMMT-1 but not in CS-99 cells (Fig. 2A). TGFβ-II also significantly increased proliferation in FUMMT-1 but not in CS-99 cells (Supplement-1). Since FUMMT-1 expressed both the TGFβR-I and TGFβR-II, we next evaluated efficacy of LY2157299 and LY2109761 in inhibiting TGFβ-Ι induced proliferation. Both LY2157299 (Fig. 2B) and LY2109761 (Fig. 2C) dose dependently inhibited TGFβ-I induced proliferation. Surprisingly in absence of exogenous TGFβ-Ι, LY2109761 but not LY2157299 dose-dependently increased proliferation. Uncoupling the effect of TGFβR-I inhibition from TGFβR-II inhibition suggests that TGFβR-II suppresses growth signals in FUMMT-1. Indeed, TGFβR-II has previously been shown to directly associate with the CyclinB/Cdc2 complex and induce G1/S phase arrest [24].

We next evaluated the effect of TGFβ-I on the migratory potential of these cell lines using the scratch migration assay (Fig. 2D). At 8h, TGFβ-I induced significant migration in both FUMMT-1 and CS-99 that was similarly and significantly inhibited upon treatment with either LY2157299 or LY2109761. Together these results suggest that canonical TGFβ signaling is functional in UCS cell lines and phosphorylation of Smad2/3 and migration can be significantly inhibited by the TGFβR-I or TGFβR-I/II inhibitor. Proliferation response to TGFβ−Ι however is distinct for FUMMT-1 and can be inhibited by the TGFβR-I inhibitor, dual receptor inhibition while successful at inhibiting TGFβ-I mediated response might also stimulate proliferation through non-canonical pathways.

**Figure 3:**
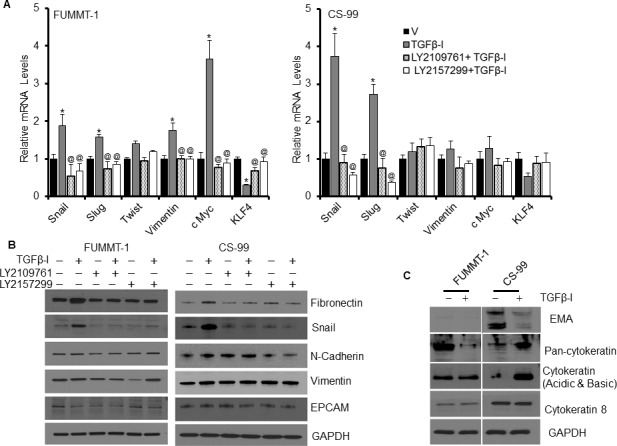
Effect of TGFβR inhibitor LY2157299 and LY2109761 on TGFβ-I induced EMT **A**. FUMMT-1 and CS-99 cell were starved, pretreated with TGFβ receptor inhibitors (5 μM) and subsequently treated with TGFβ-I (5 ng/ml) for 24 h, Total RNA was isolated and relative mRNA levels were quantified using RT-qPCR. **B.** and **C**. similarly treated cells (as in A) were lysed and processed for Western blotting. *, *P* < 0.05 was considered significant compared with vehicle and @, *P* < 0.05 was considered significant compared with TGFβ-I. Error bars represent SD.

At the mRNA level expression of Snail, Slug, Twist1, Vimentin, KLF4 and c-Myc were studied as indicators of EMT using RT-qPCR (Fig. 3A). Post TGFβ−Ι treatment, mRNA of Snail and Slug were significantly induced in both FUMMT-1 and CS-99 that could be attenuated to the basal level by the TGFβR-I or TGFβR-I/II inhibitor treatment. In addition, in FUMMT-1 post TGFβ−Ι treatment there was significant upregulation of c-Myc while KLF-4 was downregulated at the mRNA level that returned to near control levels upon treatment with either inhibitor (Fig. 3A). At the protein level, TGFβ-I induced fibronectin and Snail in both the cell lines that returned to control levels upon treatment with TGFβR-I or TGFβR-I/II inhibitor (Fig. 3B). N-Cadherin and Vimentin levels did not significantly change in either cell line after treatment with TGFβ−Ι. While E-Cadherin was not detected in these cell lines at the protein level, we focused on two other epithelial markers, Epithelial Cell Adhesion Molecule (EPCAM) and Epithelial Membrane Antigen (EMA) (Fig. 3C). Post TGFβ−Ι treatment, downregulation of EPCAM was noticeable in FUMMT-1 while downregulation of EMA was striking in CS-99 cells. An evaluation of the cytokeratin (CK) levels demonstrated no significant changes in levels of CK-8 in either cell lines. Post TGFβ−Ι treatment significant downregulation in FUMMT-1 while significant upregulation in CS-99 cells was observed using the pan-cytokeratin antibody that detects CK-1, 5, 6 and 8. On the other a second cytokeratin antibody that detects acidic and basic CKs demonstrated significant upregulation at ~50 Kda in CS-99 cells post TGFβ-I treatment (Fig. 3C). Taken together it is clear that these cell lines express both epithelial and mesenchymal components and treatment with TGFβ-I further potentiate mesenchymal differentiation evidenced by increased Slug, Snail and fibronectin expression. The significant expression of EMA in CS-99 but not in FUMMT-1, suggest a predominance of the epithelial component in this cell line. While KLF-4 is known to regulate EMT [25, 26] and its downregulation in FUMMT-1 is significant, the lack of significant KLF-4 downregulation in CS-99 suggests other molecular mechanisms such as Smad signaling to be instrumental in inducing EMT in UCS.

### TGFβ-I and c-Myc signaling in UCS

**Figure 4:**
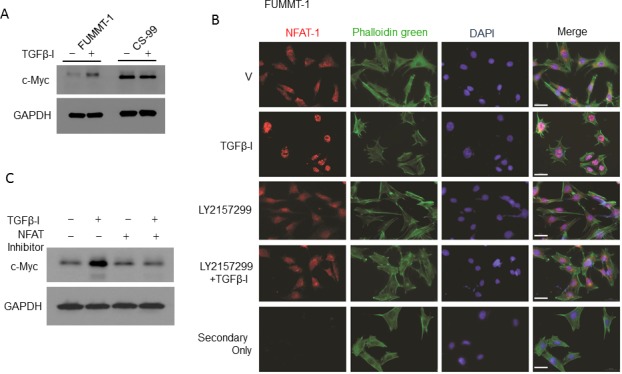
TGFβ-I mediated c-Myc expression is regulated through NFAT-1 **A**. FUMMT-1 and CS-99 cells were starved, treated with TGFβ-I (5 ng/ml) for 24h, lysed and immunoblotted for c-Myc and GAPDH. **B**. Effects of TGFβ-I and LY2157299 on NFAT-1 translocation was assayed by immunofluorescence in FUMMT-1. The images were acquired using a 40X objective (1.6X Optovar) with Zeiss Observer 2. Scale bar represents 50 um. **C**. FUMMT-1 cells were starved, pretreated with NFAT-1 inhibitor (100 μM, 1 h) subsequently treated with TGFβ-I (5 ng/ml) for 24 h, lysed and processed for Western blotting.

**Figure 5:**
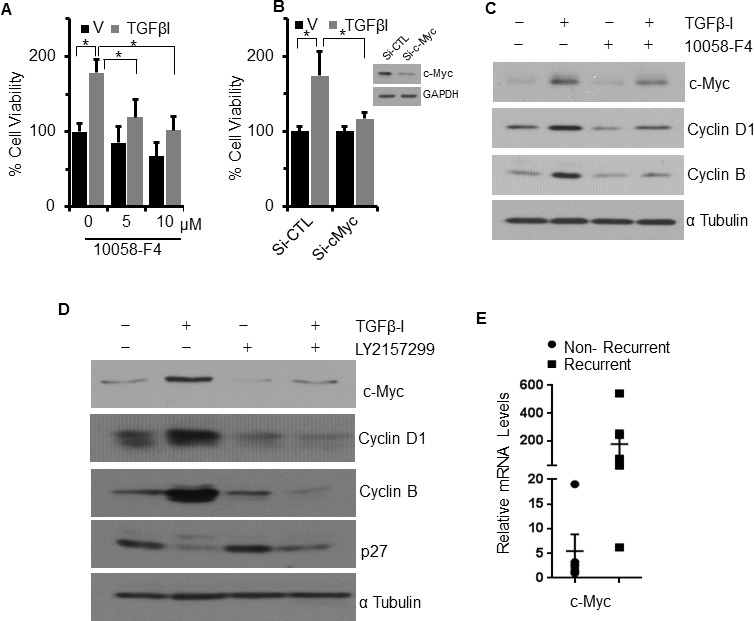
TGFβ-I induced proliferation and cell cycle is mediated through c-Myc **A**. FUMMT-1 cells were starved pretreated with c-Myc inhibitor subsequently treated with Vehicle (V) or TGFβ-I (5 ng/ml) and relative cell viability was quantified using MTS assay. **B**. FUMMT-1 cells were transfected with either non-target si-RNA (Si-CTL) or Si-c-Myc, cells were platted in 96 well plate and TGFβ-I induced proliferation was evaluated using MTS assay. Efficiency of c-Myc silencing is shown in inset. **C**. FUMMT-1 cells were starved pretreated with c-Myc inhibitor (10058-F4, 5 μM, 1 h) and treated with Vehicle (V) or TGFβ-I (5ng/ml) for 24 h lysed and processed for Western blotting. **D**. FUMMT-1 cell were starved pretreated with LY2157299 for 1 h, subsequently treated with TGFβ-I for 24 h, lysed and processed for Western blotting. **E**. RNA was isolated from UCS patient samples and relative c-Myc mRNA levels with respect to non-recurrent patient sample having highest ΔCT value were quantified using RT-qPCR, Error bars represent standard error of mean.*, *P* < 0.05 was considered significant. Error bars represent SD except where indicated.

Despite preservation of Smad signaling in both the cell lines, TGFβ−Ι induces proliferation in FUMMT-1 and not in CS-99. Strikingly TGFβ−Ι induces c-Myc mRNA and protein expression in FUMMT-1 only (Fig. 3A and 4A). Conventionally TGFβ is known to repress c-Myc transcription [27], where Smad3 binds to the TGF-β inhibitory element (TIE) on the c-Myc promoter [27]. However a previous report demonstrated that in pancreatic cancer TGFβ induces nuclear translocation of NFAT-1 that displaces Smad3 repressor complexes from the c-Myc promoter resulting in transcription and a switch from cell cycle inhibitor to growth promoter activities [28]. In corroboration we find that in FUMMT-1, TGFβ−Ι induces nuclear translocation of NFAT-1 (Fig. 4B) resulting in c-Myc expression. Interestingly LY2157299 treatment blocked nuclear translocation of NFAT-1 (Fig. 4B). Using the NFAT inhibitor we showed that TGFβ−Ι mediated expression of c-Myc was NFAT-1 dependent (Fig. 4C). Furthermore the TGFβ−Ι induced proliferation response in FUMMT-1 was c-Myc dependent and could be attenuated by using a pharmacological (10058-F4) [29] (Fig. 5A) or genetic inhibitor (siRNA) of c-Myc (Fig. 5B). The c-Myc dependent proliferation was likely mediated through activation of cell cycle as evidenced by a significant increase in Cyclin B [30] and Cyclin D1 [31] protein levels that could be attenuated by c-Myc inhibitor (Fig. 5C) or c-Myc siRNA treatment (Supplement-2). Finally the TGFβR-I inhibitor also significantly downregulated TGFβ−Ι induced c-Myc, Cyclin B and Cyclin D1 levels while upregulating the cell cycle inhibitor p27 [32] (Fig. 5D). Interestingly the mRNA levels of c-Myc were elevated in the recurrent compared to the non-recurrent group of UCS patient samples as well (Fig. 5E).

## DISCUSSION

Here the role of TGFβ signaling was evaluated in UCS patient samples and cell lines and efficacy of TGFβR inhibitors was determined. Though limited in number the UCS patient samples demonstrated that those with recurrent disease had a trend towards increased expression of the TGFβ signaling components with TGFβ-1 and TGFβR-II being statistically significant. The two UCS cell lines FUMMT-1 and CS-99 also expressed components of the TGFβ pathway. More importantly, TGFβ−Ι induced canonical activation of Smad2 and Smad3 in both of these cell lines that could be attenuated by LY2157299, TGFβR-I or LY2109761, the TGFβR-I and II dual inhibitor.

In keeping with the biphasic nature of UCS [33, 34], these cell lines though predominantly sarcomatous reportedly express epithelial components [35]. Clinically, the epithelial component of USC is usually immunoreactive with cytokeratins, EMA, and vimentin. The mesenchymal component usually stains for vimentin, smooth muscle actin, desmin, and focal cytokeratin [36]. Both the sarcomatous and carcinomatous components often coexpress epithelial markers and vimentin to varying degrees [33, 37, 38]. While TGFβ signaling is established to be anti-proliferative in epithelial cells [10, 39], its role in mesenchymal cells remains ambiguous. Our data clearly indicated that both the cell lines responded to TGFβ by upregulating mesenchymal markers such as Snail, Slug and fibronectin that could be attenuated by LY2157299 or LY2109761. However distinct differences in cytokeratin expression and its regulation in response to TGFβ−Ι were observed in the two cell lines. Also only CS-99 expressed EMA that was significantly downregulated by TGFβ−Ι suggesting that the epithelial component might be more prominent in this cell line compared to FUMMT-1. In addition, both the cell lines demonstrated significant migration in response to TGFβ−Ι that could be significantly inhibited by LY2157299 or LY2109761. Hence our data demonstrate that TGFβ−Ι induced Smad activation, EMT and migration response could be attenuated by inhibiting either TGFβR-I or TGFβR-I/II.

TGFβ however could potentiate proliferation only in the cell line that also induced c-Myc. Though conventionally TGFβ signaling inhibits c-Myc transcription, we believe in the mesenchymal component of UCS, TGFβ induces nuclear translocation of NFAT-1 that displaces Smad3 repressor complexes leading to c-Myc transcription. In corroboration, a previous report demonstrated that in pancreatic cancer TGFβ induced nuclear translocation of NFAT-1 that displaced Smad3 repressor complexes from the c-Myc promoter resulting in transcription and a switch from cell cycle inhibitor to growth promoter activities [28]. Enhanced c-Myc expression then activates cell cycle leading to unrestricted proliferation, all of which can be readily attenuated by the TGFβR-I inhibitor. Curiously in absence of exogenous TGFβ−Ι, the dual inhibition of TGFβR-I and II but not TGFβR-I increases proliferation dose-dependently. This suggests that in absence of TGFβ−Ι, the function of the two receptors can be uncoupled. TGFβR-II functions as an inhibitor of pro-proliferative pathways that most likely do not involve Smads. Indeed TGFβR-II has previously been shown to directly associate with the CyclinB/Cdc2 complex and induce G1/S phase arrest [24].

In conclusion these data demonstrate that the TGFβ pathway is functional in UCS and instrumental in inducing EMT, migration and proliferation, all of which can be potently inhibited by the TGFβR-I inhibitor, LY 2157299. Galunisertib or LY 2157299 is currently the only TGFβR-I kinase inhibitor that is in phase II clinical trials for glioma, pancreatic cancer, myelodisplastic syndrome and hepatocellular cancer. Therefore inhibition of TGFβR-I could be efficacious in treatment of UCS and NFAT-1 and c-Myc could be potential markers predicting poor outcome.

## MATERIALS AND METHODS

### Cell culture

The human UCS cell line FUMMT-1 was purchased from Dr. Makoto Emoto (Division of Gynecology, Center of Preventive Medicine, Fukuoka Sanno Hospital, International University of Health and Welfare, Japan) and CS-99 was a kind gift from Dr. Jason Somarelli; Duke University Medical Center [upon approval from Dr. H. J. Schulten; (Institute of Pathology, University of Göttingen, Germany) and Dr. Gloria Huang (The University Hospital for Albert Einstein College of Medicine)].. FUMMT-1 was cultured in DMEM-F12 and CS-99 in DMEM supplemented with 10% heat inactivated FBS (Fisher Scientific) and 100 units penicillin and 100 μg streptomycin/ml (Invitrogen).

### Tumor samples

In this study, 10 UCS patient samples were acquired from the Stephenson Cancer Center Biospecimen Acquisition Core and Bank following institutional guidelines. Of the 10 patients, 5 have recurred (4 are dead, one remains alive at 22 mo following salvage radiation and chemotherapy for a vaginal cuff recurrence), and 5 remain free of recurrence (follow-up time ranges from 5-60 mo). The median PFS was 4.8 (range 3-7 mo) for the recurrent group.

### Cell viability assay

Viability of UCS cells was determined using the tetrazolium compound based CellTiter 96^®^ AQ_ueous_ One Solution Cell Proliferation (MTS) assay (Promega). Cells were plated in 96 well plate at density of 2.5x 10^3^ cells per well. 4h post serum starvation cells were subjected to TGFβ-I (R&D Biosystems) treatment alone or in presence of LY2157299 [40] (TGFβR-I inhibitor, Selleck Chem), LY2109761 [41] (TGFβR-I/II inhibitor, Selleck Chem), 10058-F4 (c-Myc Inhibitor, Sigma Aldrich), NFAT inhibitor (Cay-man Chemicals). After 24h treatment MTS assay was done and viability was calculated using vehicle as 100%. [42].

### TGFβ-I ELISA

TGFβ-I secretion was measured using Quantikine human TGFβ-I ELISA kit (R&D Systems) following the manufacturer's direction. Briefly equal number of FUMMT-1 and CS-99 cells were plated on 60 mm plate, were serum starved and supplemented with serum-free medium for 48 h. Cell-free supernatant was collected and total TGFβ-I was quantified by interpolation from the standard curve generated with rTGFβ-I. Data are reported as the mean level of triplicate samples ± SD normalized with total protein content of cells.

### RNA isolation and RTqPCR

Total RNA from frozen patient tumor samples and cell lines were extracted using quick RNA miniprep kit (Zymo Research) following manufacturer's protocol. 1μg of isolated RNA was reverse transcribed (iScript cDNA Synthesis kit, Bio-Rad) using random hexamer primers. RT-qPCR analysis was performed using iTaq™ universal SYBR® Green supermix. Primers were designed using Primer-BLAST and synthesized from Integrated DNA Technologies (IDT, sequences are available upon request). The comparative ΔΔC_t_ method was used to calculate the relative abundance of the mRNA and normalized with that of 18s rRNA [43] and western blotting.

### siRNA transfection

Gene silencing was performed in 60 mm culture dish containing 5×10^5^ cells in suspension using Hiperfect (Qiagen) and 100nM siRNA (scrambled control, Dharmacon) Si-c-Myc (Sigma) in OPTIMEM (Invitrogen). Effective silencing was achieved after 48h of transfection determined by RT-qPCR [44].

### Western blotting

Total cell lysate was prepared in RIPA (Boston Bioproducts) containing protease and phosphatase inhibitor cocktail (Thermo). The cell lysate was quantified and proteins separated on SDS-PAGE and transferred to the PVDF (Bio-Rad) membrane. Membranes were blocked in 5% nonfat milk in TBS with 0.1% Tween-20 (TBST) for 1 h at room temperature followed by incubation with primary antibodies in TBST with 1% BSA overnight. The following primary antibodies were used – p Smad-2, p Smad-3, Smad-2, Smad-3, Snail, Vimentin, EPCAM, c-Myc, NFAT-1, Cyclin B, Cyclin D1, (Cell signaling Technology), Fibronectin, N-Cad (BD Biosciences), Pancytokeratin, GAPDH, α tubulin and β actin (Sigma), Acidic and basic Cytokeratin AE1/AE3 (Millipore), EMA (Dako), p27 (Santa Cruz Biotechnology), and cytokeratin-8 (Developmental Studies Hybridoma Bank, University of Iowa). Secondary antibodies (from sigma) were used at a concentration of 1:10,000. Equal loading was verified by immunoblotting with GAPDH, αTubulin or β Actin [44].

### Migration assay

TGFβ-I induced migration was evaluated using the scratch migration assay. FUMMT-1 and CS-99 cells were plated in 6-well plate and were allowed to grow in 10% FBS containing medium to confluence. Cells were then serum starved for 4 h and treated with TGFβR inhibitors (5μM). 1h post inhibitor treatment cells were scratched with a pipette tip, washed twice with PBS and treated with either vehicle, TGFβ-I 5 ng/ml or TGFβR inhibitor + TGFβ-I in media containing 0.5% FBS. Cells were imaged immediately and 8 hours after treatment using an Olympus CK40 microscope. Number of migrated cells from 3 different fields were counted using ImageJ analysis software (NIH) [44]. Experiment was repeated three times in triplicate.

### Immunofluorescence

Localization of NFAT-1 was determined by immunostaining followed by microscopy. Approximately, 1.5×10^3^ cells were plated per chamber of 4-chambered slides. After 18 h, the cells were washed twice with serum-free medium, serum starved and treated as indicated. 6h post TGFβ-I (5ng/ml) treatment cells were fixed with 4% PFA, permeabilized with 0.25% TritonX-100 in PBS, blocked with 5% BSA in PBS, incubated with primary NFAT-1 antibody in 1% BSA-PBS (overnight), followed by incubation with the goat anti-rabbit secondary (Alexa flour 568, 1:1000 dilution in 1% BSA containing PBS) and Phalloidin-Alexa 488 conjugate (Invitrogen; 1:500 dilution in 1% BSA containing PBS) for 1h at RT. The cells were washed 3×3 min with PBS after each step during the immunostaining. The cells were then mounted with VECTASHIELD^®^ mounting medium with DAPI (Vector Laboratories). The images were acquired using 40X objective (with 1.6X Optovar) using Zeiss Observer 2.

### Data analysis and statistics

All the experiments were performed in triplicate and repeated independently 3 times. Transfection experiments were performed by pooling cells from 2 different independent transfection, which were again performed in duplicate. Data are expressed as means ± standard deviation (SD) or standard error of the mean (SEM) as indicated. Student's t test was used for statistical analysis with significance set at P < 0.05.

## SUPPLEMENTARY MATERIAL FIGURES


