# Supplementary material for: Role of TGF-β signaling in uterine carcinosarcoma
Source: Oncotarget. 2015 Mar 30;6(16):14646–55. doi: 10.18632/oncotarget.3711 (PMC4546494; doi:10.18632/oncotarget.3711)
Supplement: Supplementary file 1 [file oncotarget-06-14646-s001.pdf]

Role of TGF-β signaling in uterine carcinosarcoma

Supplementary Material

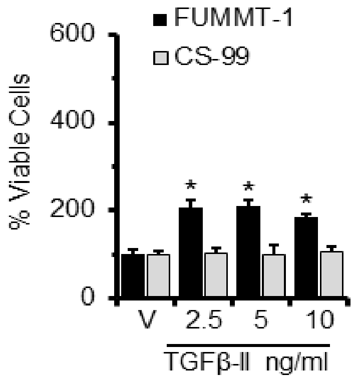

Supplemental Figure 1: TGFβ-II mediated proliferation in UCS cell lines.

Cells were serum starved and treated with TGFβ-II for 24 h, % cell viability was calculated using MTS assay *, $P < 0.05$ was considered significant. Error bars represent SD.

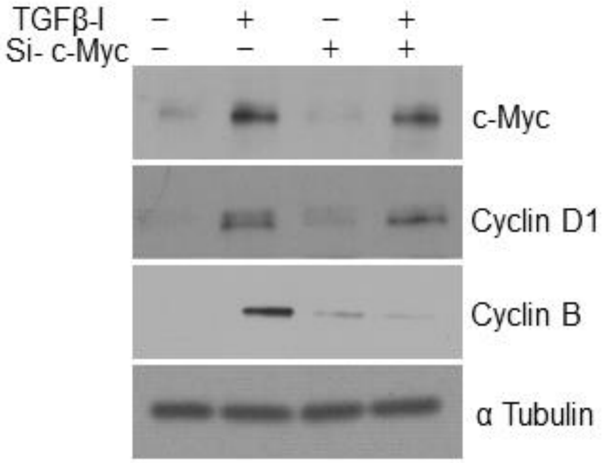

Supplemental Figure 2: TGFβ-I induced cell cycle progression is mediated through c-Myc.

FUMMT-1 cells were transfected with either non-target si-RNA (Si-CTL) or Si-c-Myc, 48h post transfection cells were serum starved (4h) subsequently treated with TGFβ-I or vehicle for 24h, lysed and processed for Western blotting.
